# miRNA‐532‐5p functions as an oncogenic microRNA in human gastric cancer by directly targeting RUNX3

**DOI:** 10.1111/jcmm.12706

**Published:** 2015-10-30

**Authors:** Xia Xu, Yingjie Zhang, Zhifang Liu, Xinchao Zhang, Jihui Jia

**Affiliations:** ^1^ Department of Biochemistry and Molecular BiologySchool of MedicineShandong UniversityJinanChina; ^2^ Department of Radiation OncologyShandong Cancer HospitalJinanChina; ^3^ Department of MicrobiologyKey Laboratory for Experimental Teratology of Chinese Ministry of EducationSchool of MedicineShandong UniversityJinanChina

**Keywords:** miR‐532‐5p, RUNX3, carcinogenesis, gastric cancer

## Abstract

Accumulating data reveal that microRNAs are involved in gastric carcinogenesis. To date, no information was reported about the function and regulatory mechanism of miR‐532‐5p in human gastric cancer (GC). Thus, our study aims to determine the role and regulation of miR‐532‐5p in GC. Here, we found that transient and stable overexpression of miR‐532‐5p dramatically increased the potential of colony formation and migration of GC cells, decreased the percentage of cells in G1 phase and cell apoptosis *in vitro*, and increased the weight of mice lungs and number of lung xenografts *in vivo*. Gain‐of‐function, loss‐of‐function and luciferase activity assays demonstrated that miR‐532‐5p negatively regulated the expression of RUNX3 and its targets directly. We also found that miR‐532‐5p level was negatively correlated with RUNX3 gene expression in various GC cell lines. Our results indicate that miR‐532‐5p functions as an oncogenic miRNA by promoting cell growth, migration and invasion in human GC cells.

## Introduction

Gastric cancer is one of the most common human malignant tumours and ranks the second in terms of global cancer‐related death 1. Clinically, the absence of specific symptoms renders early diagnosis of this deadly disease difficult, thus most of patients are diagnosed at advanced stages. Improvement of diagnosis and treatment has resulted in good long‐term survival for patients with early GC, whereas the prognosis of patients with advanced GC is still poor 2. Therefore, further studies are necessary to better understand the pathogenesis of GC. Recent studies have revealed that microRNAs (miRNAs) are novel regulator of tumour progression and potential therapeutic targets in GC 3, 4.

miRNAs are small, single‐strand, 18–25 nucleotides RNAs resulting in target mRNA degradation or translational repression 5. Accumulating evidence indicate that miRNAs are involved in many physiological processes, including cellular proliferation, differentiation, development and apoptosis 6, 7. Clear explanation of miRNAs function and regulation of their targets will bring a prospective future for diagnosis and treatment of GC.

miR‐532‐5p is located at human chromosome Xp11.23 and mature miR‐532‐5p consists of 22 nucleotides. Analysis of miR‐532‐5p sequence by ClustalW displays its conservative sequence between various species, which implies its important role in evolutional progress. However, little information was reported about miR‐532‐5p. We tried to get some useful information using different algorithms. At least four databases including miRBase, TargetScan, microrna.org and Diana demonstrate that the 2–8 bases (seed sequence) and 14–20 bases of miR‐532‐5p are completely complementary with the 890–908 bases of known tumour suppressor gene RUNX3 mRNA 3′‐UTR, indicating that RUNX3 is a potential target of miR‐532‐5p. Moreover, there is no report about the function of miR‐532‐5p in human GC, and our group focused on RUNX3 in human GC 8, 9, 10. Accordingly, our study aims to determine the role of miR‐532‐5p in tumorigenesis and progression of GC and regulation of RUNX3 by miR‐532‐5p.

## Materials and methods

### miR‐532‐5p mimics, inhibitor, plasmid and cell transfection

The specific miR‐532‐5p mimics and control mimics, miR‐532‐5p inhibitor and control inhibitor were purchased from RiboBio (Guangzhou, China). The miR‐532‐5p eukaryotic expression plasmid p*Silencer* ^™^ 4.1‐CMV‐532 was constructed in this study. Fugene^®^ HD transfection reagent (Roche, Mannheim, Germany) and Lipofectine 2000 (Invitrogen, Carlsbad, CA, USA) were used to transfect the plasmid and miR‐532‐5p mimics or inhibitor into the GC cells respectively.

### Cell culture and stable BGC‐823 cell

Human GC cells AGS, BGC‐823, SGC‐7901, HGC‐27, KATO‐III, MKN‐45, NUGC‐3 and immortalized GES‐1 were maintained in our laboratory. AGS was cultured in F12 medium (Gibco BRL, Gaithersburg, MD, USA) with 10% foetal bovine serum (FBS). Other cells was cultured in RPMI 1640 medium (Invitrogen) with 10% FBS. All cells were cultured at 37°C/95% air/5% CO_2_ in a humidified incubator.

After transfecting p*Silencer* ^™^ 4.1‐CMV‐532 into BGC‐823 cells, the stable cell clones were selected by resistance to G418 followed by limited dilution cloning. The relative control clones were produced by the similar methods. The high level of miR‐532‐5p in stable cells was confirmed by Quantitative RT‐PCR (QRT‐PCR).

### RNA extraction, reverse transcription and QRT‐PCR

Total RNAs from cells or tissues were extracted using Trizol reagent (Invitrogen) according to the manufacturer's instructions. Reverse transcription of RNAs was performed with M‐MLV reverse transcriptase (Fermentas, Vilnius, Lithuania). cDNA for miRNA and total cDNA were synthesized using the specific miRNA primers from RiboBio and random hexamer from Fermentas respectively. Expression of RUNX3 mRNA and mature miR‐532‐5p were assessed by QRT‐PCR using SYBR^®^ Premix Ex Taq^™^ (TaKaRa, Dalian, China). QRT‐PCR was performed in an ABI7700 sequence detector (Applied Biosystems, Foster City, CA, USA). Beta‐2‐microglobulin and U6 small nuclear RNA were used as the control for RNA loading in detection of RUNX3 mRNA and miR‐532‐5p respectively.

### Western blotting

Total cellular proteins were extracted with lysis buffer, separated by SDS‐PAGE and transferred to a nitrocellulose membrane. After incubating in the blocking buffer, the membrane was probed with specific antibodies against RUNX3, Bim (both from Abcam, Cambridge, UK), p21 or β‐actin (both from Santa Cruz Biotechnology, Santa Cruz, CA, USA) overnight at 4°C, followed by horseradish peroxidase‐conjugated IgG and developed with enhanced chemiluminescent reagent (Millipore, Billerica, MA, USA) according to the manufacturer's instructions.

### Construction of reporter plasmid and luciferase activity assay

The 315 bp wild‐type 3′‐UTR of human RUNX3 mRNA containing miR‐532‐5p binding site was amplified by PCR and inserted into the SpeI/HindIII sites of pMIR‐REPORT^™^ luciferase reporter plasmid to generate pMIR‐RUNX3/wt plasmid. The complementary sequence for miR‐532‐5p seed sequence in RUNX3 3′‐UTR was mutated using QuickChange site‐directed mutagenesis kit with the pMIR‐RUNX3/wt plasmid as template. The mutant was named as pMIR‐RUNX3/mut plasmid. The cells were cotransfected with miR‐532‐5p mimics and pMIR‐RUNX3/wt or pMIR‐RUNX3/mut transiently. Meanwhile, pMIR‐REPORT^™^ β‐gal control plasmid was cotransfected to normalize variability because of differences in cell viability and transfection efficiency. 48 hrs later, luciferase activity and β‐galactosidase activity were determined using a dual luciferase reporter assay system and β‐Galactosidase enzyme assay system (both from Promega, Madison, WI, USA).

### Colony formation assay

The cells were seeded into 6‐well plates (300 cells/well) and cultured for 10–14 days at 37°C with 95% air/5% CO_2_. Cell colonies were fixed with methanol and stained with crystal violet, and the colony foci with more than 50 cells were counted.

### Cell cycle and apoptosis assay

For cell‐cycle assay, the cells were fixed with 70% ethanol and stained with propidium iodide (PI) containing RNase A in the dark. For apoptosis assay, the cells were stained with PI and AnnexinV‐FITC. Thereafter, the cells were analysed using a flow cytometer (BD Biosciences, Bedford, MA, USA). Each experiment was performed in triplicate and the data were analysed with FCS Express V3.0612 soft (De Novo Software, Glendale, Canada).

### Cell migration assay

The cells were harvested and resuspended in serum‐free RPMI 1640 medium, and 3 × 10^4^ cells were placed into chambers (Costar, Cambridge, MA, USA). The chambers were then inserted into the wells of 24‐well plates and incubated. After 48 hours' culture, the cells remaining on the upper surface of the chamber were gently removed, whereas the cells adhering to the lower surface were fixed with methanol, stained with crystal violet and counted under a microscope (Olympus, Shinjuku, Tokyo, Japan).

### Nude mice xenograft model

The lung tumour xenografts were established by injecting 6 × 10^5^ stable cells into the tail vein of athymic 5–6 week‐old BALB/c nude mice (Peking University, Beijing, China). The mice were killed after 4 weeks and the dissected lungs were collected and weighed. A part of lung tissues were fixed in paraformaldehyde, embedded in paraffin, sectioned at 4 μm thickness and stained with haematoxylin–eosin. The rest of lung tissues were used to examine the expression of miR‐532‐5p and RUNX3. All the animal experiments were approved by the local ethics committee of Shandong University.

### Statistical analysis

All data presented were repeated at least three independent experiments. Student's *t*‐test was used to evaluate the data of different treatment group and *P* < 0.05 was considered statistically significant. All data were analysed by SPSS17.0 statistical software (SPSS Inc., Chicago, IL, USA).

## Result

### miR‐532‐5p promotes GC cells growth and migration *in vitro*

**Figure 1:**
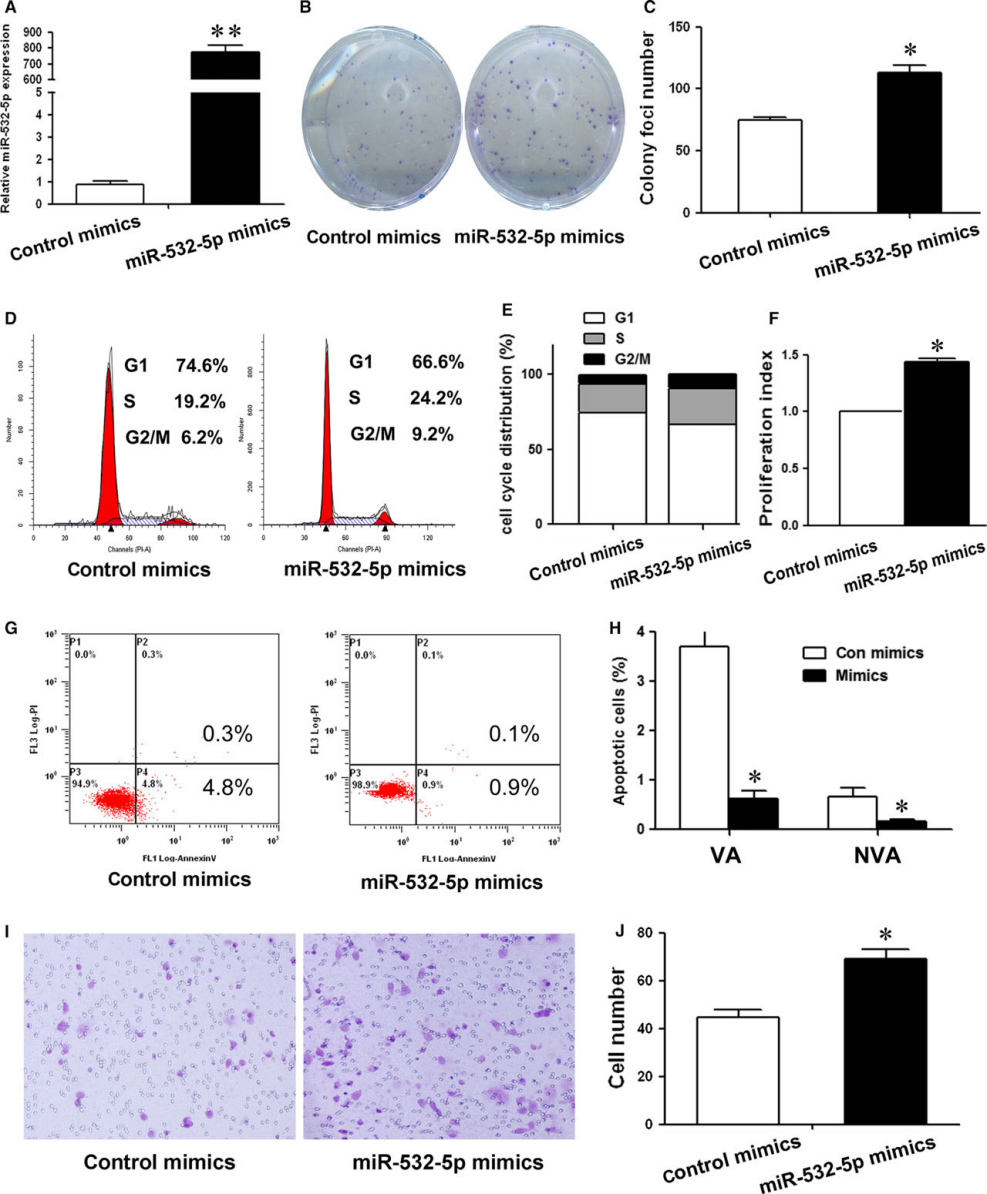
The function of miR‐532‐5p in transiently transfected BGC‐823 cells. (**A**) miR‐532‐5p expression in transfected cells was determined by QRT‐PCR. (**B**) The clonogenic potential was assessed. The representative results were shown. (**C**) The number of colony foci was counted and analysed by Student's *t*‐test. (**D**) Distribution of cells in three phases of cell cycle was examined by flow cytometry assay. (**E**) Cytometric quantification in (**D**) showed the proportion of cells in G0/G1, S and G2/M phase. (**F**) The proliferation index (PI) was calculated using the following equation: PI = (S+G2)/G1, where S, G2 and G1 are the ratio of cells in S, G2/M and G0/G1 phase respectively. (**G**) Apoptotic cell was examined by flow cytometry assay. (**H**) The ratio of viable apoptotic cells (VA) and non‐viable apoptotic cells (NVA) in (**G**) was analysed statistically. (**I**) The migration potential was assessed by transwell assay. (**J**) The number of cells adhering to the lower surface of the chamber was counted and analysed by Student's *t*‐test (*P < 0.05, **P < 0.01).

**Figure 2:**
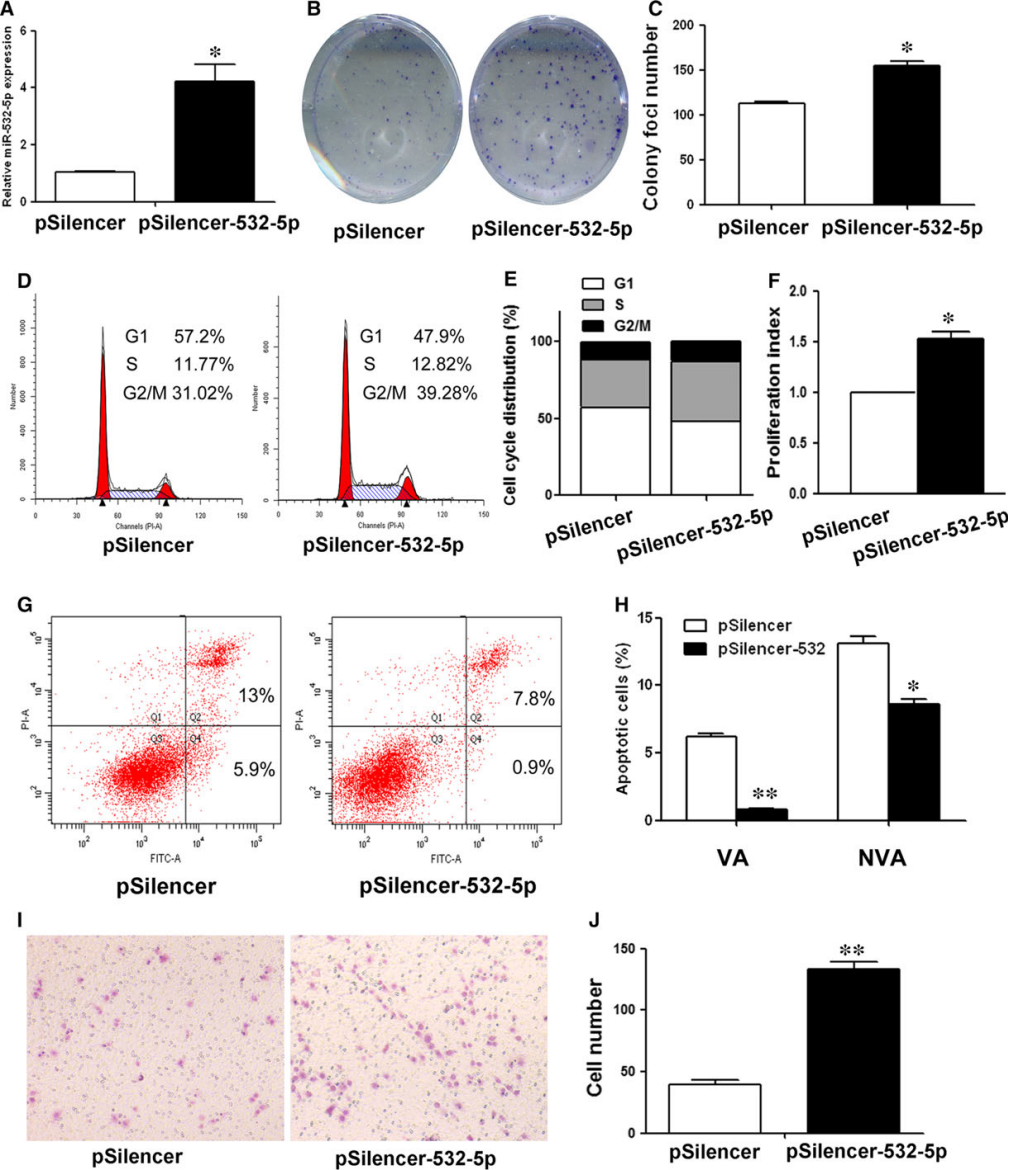
The role of miR‐532‐5p in stable miR‐532‐5p expressing BGC‐823 cells. (**A**) miR‐532‐5p level in stable cells was determined by QRT‐PCR. (**B**) The clonogenic potential was assessed. The representative results were shown. (**C**) The number of colony foci was counted and analysed by Student's *t*‐test. (**D**) Distribution of cells in three phases of cell cycle was examined by flow cytometry assay. (**E**) Cytometric quantification in (**D**) showed the percentage of cells in G0/G1, S and G2/M phase. (**F**) The proliferation index (PI) was calculated using the following equation: PI = (S+G2)/G1, where S, G2 and G1 are the ratio of cells in S, G2/M and G0/G1 phase respectively. (**G**) Apoptotic cell was examined by flow cytometry assay. (**H**) The percentage of viable apoptotic cells (VA) and non‐viable apoptotic cells (NVA) in (**G**) was analysed statistically. (**I**) The migration potential was assessed by transwell assay. (**J**) The number of cells adhering to the lower surface of the chamber was counted and analysed statistically (*P < 0.05, **P < 0.01).

To determine the functions of miR‐532‐5p in human GC cells, several experiments were performed. The specific miR‐532‐5p mimics was used to reinforce miR‐532‐5p expression (Fig. 1A). Stable cell clone was established by G418 selection and exhibited high miR‐532‐5p expression compared to control (Fig. 2A). Colony formation assay showed that both transient and stable overexpression of miR‐532‐5p led to an increase in foci number as well as size in GC cells (Figs 1B, C and 2B, C), indicating that miR‐532‐5p promoted cell growth. To explore the mechanisms underlying the promotion of cell growth, we examined cell cycle distribution and apoptosis after miR‐532‐5p treatment. Both transient and stable overexpression of miR‐532‐5p decreased cell number in G1 phase, which was accompanied by an increase in the cell population in S phase and G2/M phase (Figs 1D, E and 2D, E). Compared to negative control, the proliferation index of miR‐532‐5p‐treated cells was higher (Figs 1F and 2F). Moreover, overexpressed miR‐532‐5p suppressed cell apoptosis (Figs 1G and 2G). Both viable apoptotic cells and non‐viable apoptotic cells decreased significantly after miR‐532‐5p treatment (Figs 1H and 2H). Lastly, miR‐532‐5p overexpression induced more number of GC cells to migrate through the chamber well adhering to the low surface (Figs 1I, J and 2I, J). These results indicate that miR‐532‐5p functions as an oncogenic miRNA by promoting GC cell growth by pushing cell cycle progression and inhibiting cell apoptosis, and promoting GC cells migration.

### miR‐532‐5p promotes GC cells invasion and colonization *in vivo*

**Figure 3:**
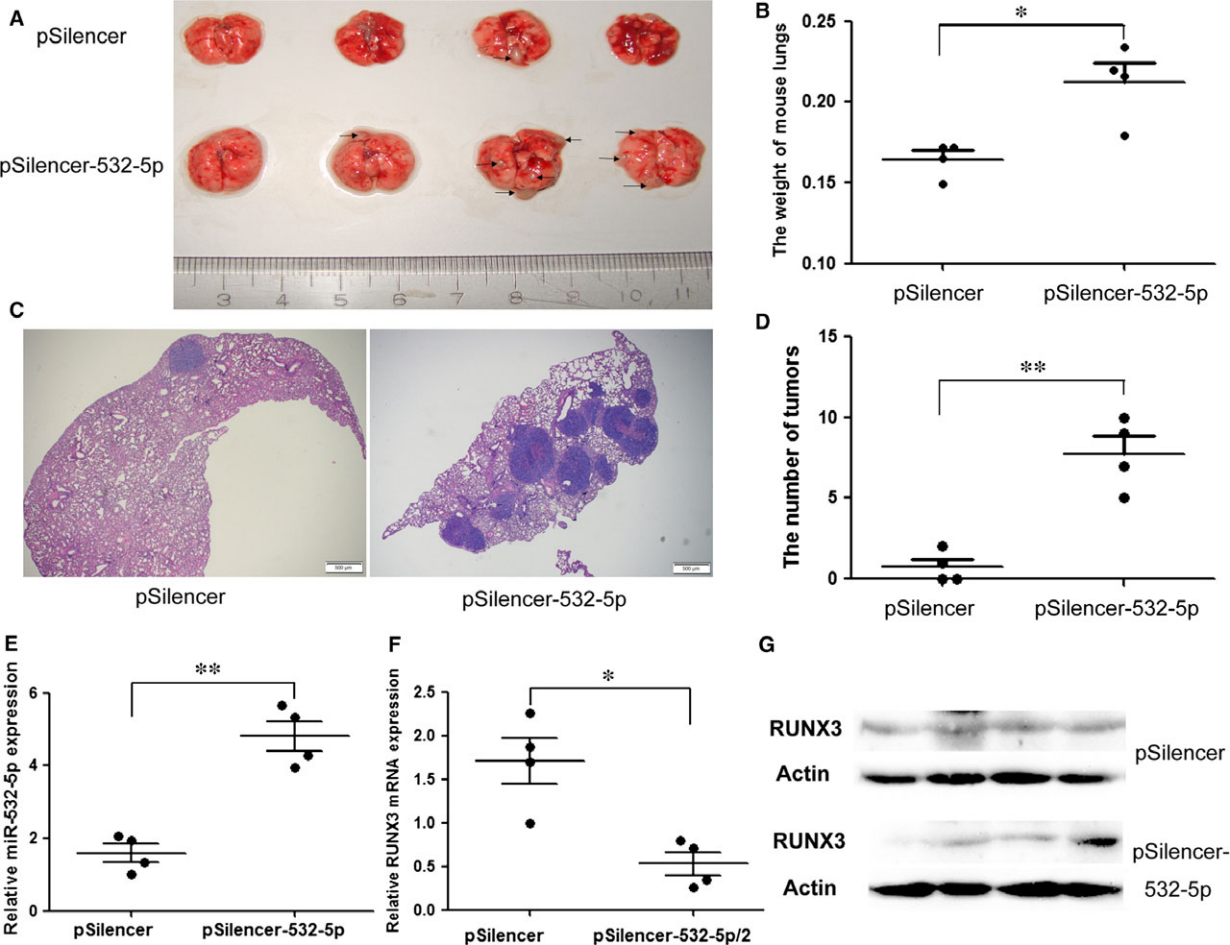
The role of miR‐532‐5p in nude mice with stable miR‐532‐5p expressing BGC‐823 cells. (**A**) After 4 weeks' treatment with stable BGC‐823 cells, the lung xenografts were developed and the lungs were dissected (The scale bar indicates the lungs size and the black arrows denote tumour nodules). (**B**) The weight of lungs was assessed and analysed statistically. (**C**) The lung tissue slices were stained with HE. The representative results were shown (The scale bar represents 500 μm and the dark blue nodule represents tumour foci). (**D**) The number of tumour foci in lungs was counted and analysed by Student's *t*‐test. (**E**–**G**) The level of miR‐532‐5p, RUNX3 mRNA and protein level in lungs were examined by QRT‐PCR and western blotting respectively (*P < 0.05, **P < 0.01)

To determine whether miR‐532‐5p could trigger tumour growth *in vivo*, as observed in cultured cells, the lung xenografts were established in nude mice. The lungs of stable miR‐532‐5p expression group had a significant larger volume and weight than those of control group (Fig. 3A and B). HE staining of lung tissue slices displayed more number of tumour foci in stable miR‐532‐5p expression group (Fig. 3C and D), indicating that miR‐532‐5p facilitated tumour cell colonization and growth. Stable miR‐532‐5p expression group had higher level of miR‐532‐5p than control group (Fig. 3E), whereas RUNX3 gene expression level in stable miR‐532‐5p expression group was lower than control group (Fig. 3F and G). These data imply that miR‐532‐5p triggers GC cells invasion from the vein to lungs and strengthens GC cells colonization and growth in mice lungs.

### miR‐532‐5p inhibits RUNX3 gene expression at transcriptional and translational level

Since miRNAs exerted their functions by negatively regulating the expression of their target genes, putative targets of miR‐532‐5p were predicted using four databases. These databases predict that RUNX3 is a potential target of miR‐532‐5p. Our group has been interested in RUNX3 and there is still no report that RUNX3 is a direct target in GC, thus we investigated whether miR‐532‐5p is capable of regulating endogenous RUNX3 expression.

**Figure 4:**
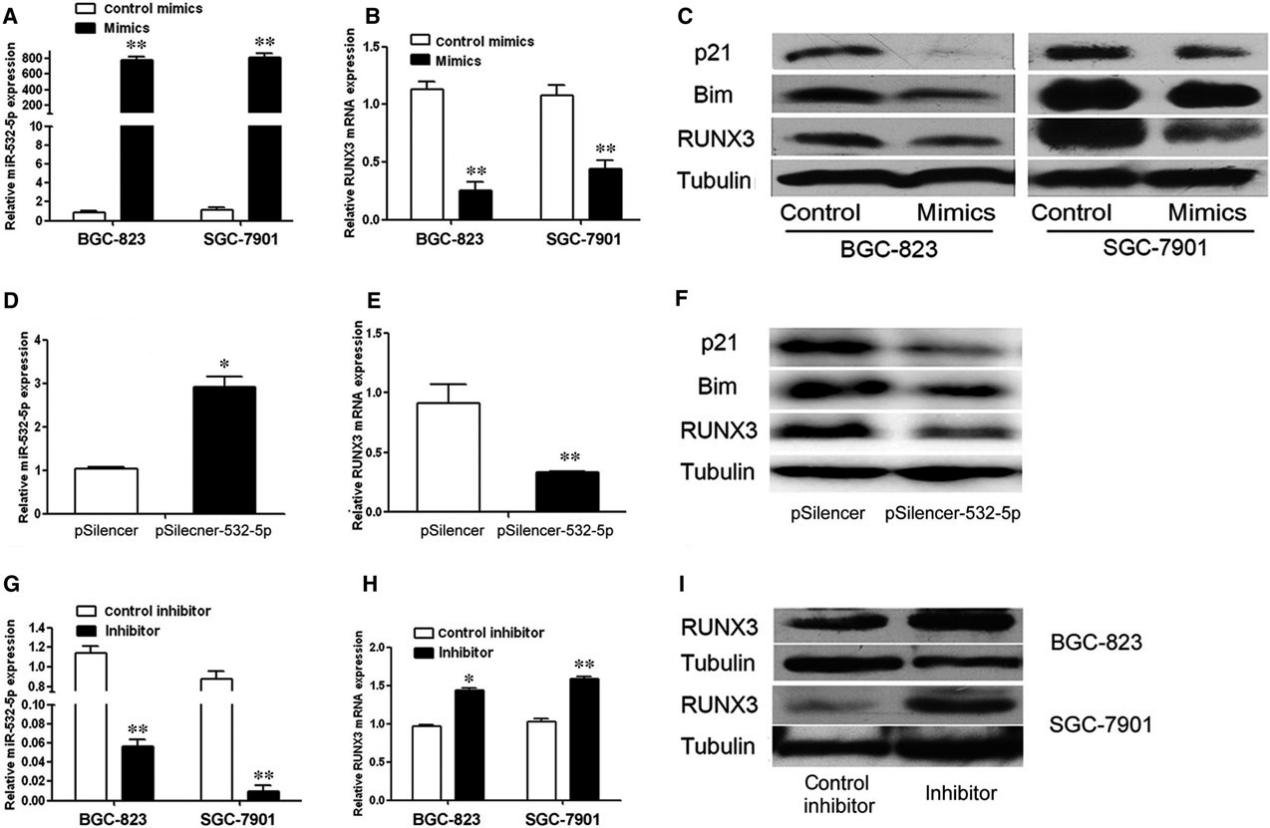
The inhibition of RUNX3 expression by miR‐532‐5p *in vitro*. (**A** and **B**) Analysis of miR‐532‐5p and RUNX3 mRNA expression in miR‐532‐5p mimics‐treated GC cells by QRT‐PCR. (**C**) Western blotting analysis of RUNX3 and its targets protein expression in miR‐532‐5p mimics‐treated GC cells. (**D** and **E**) QRT‐PCR analysis of miR‐532‐5p and RUNX3 mRNA expression in stable miR‐532‐5p expressing BGC‐823 cells. (**F**) Western blotting analysis of RUNX3 and its targets protein expression in stable BGC‐823 cells. (**G**–**I**) Analysis of miR‐532‐5p and RUNX3 gene expression in miR‐532‐5p inhibitor‐treated GC cells by QRT‐PCR and western blotting (*P < 0.05, **P < 0.01).

Gain‐of‐function and loss‐of‐function experiments were used to testify our prediction. Transfection of specific miR‐532‐5p mimics induced strong increase in mature miR‐532‐5p up to seven hundred times compared to control (Fig. 4A), which caused a sharp reduction in RUNX3 mRNA and protein expression in these cells (Fig. 4B and C). Stable miR‐532‐5p expression exhibited the similar results accordant with the mimics treatment (Fig. 4D–F). Moreover, overexpression of miR‐532‐5p resulted in the protein reduction in known RUNX3 targets such as p21 and Bim (Fig. 4C and F). On the other hand, knockdown of miR‐532‐5p expression by specific miR‐532‐5p inhibitor increased RUNX3 gene expression (Fig. 4G–I). Accordingly, miR‐532‐5p is able to suppress RUNX3 mRNA and protein expression.

### miRNA‐532‐5p expression is negatively correlated with RUNX3 gene expression in various GC cells

**Figure 5:**
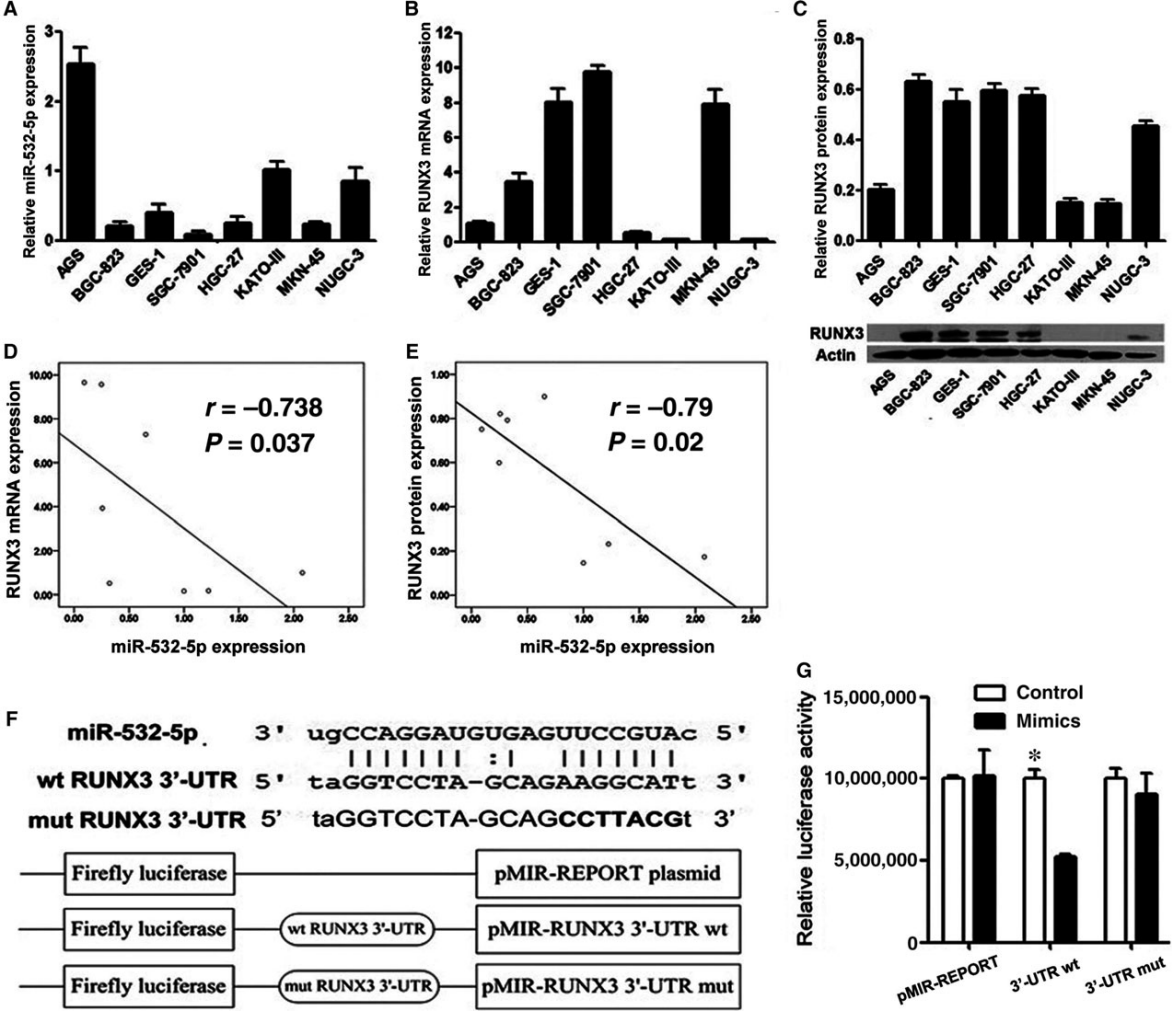
The negative RUNX3 regulation by miR‐532‐5p. (**A** and **B**) QRT‐PCR analysis of miR‐532‐5p and RUNX3 mRNA expression in various GC cell lines. (**C**) Western blotting analysis of RUNX3 protein expression in same cell lines and quantification of RUNX3 protein. (**D** and **E**) Statistical analysis of correlation of miR‐532‐5p and RUNX3 mRNA, miR‐532‐5p and RUNX3 protein by SPSS 17.0 respectively. (**F**) Upper: the predicted conserved sites for miR‐532‐5p binding in wild‐type RUNX3 3′‐UTR and mutant RUNX3 3′‐UTR (The letters in bold are mutant bases). Lower: diagram of wild‐type and mutant RUNX3 3′‐UTR‐containing reporter construct. (**G**) Luciferase activity analysis on the presence of wild‐type or mutant RUNX3 3′‐UTR and miR‐532‐5p mimics (*P < 0.05).

In addition, the expression of miR‐532‐5p, RUNX3 mRNA and protein in eight GC cell lines were examined by QRT‐PCR and western blotting respectively (Fig. 5A–C). The correlation between the level of miR‐532‐5p and RUNX3 was analysed by SPSS 17.0 statistical software. As Figure 5D and E shown, both RUNX3 mRNA and protein level were negatively correlated with the expression of miR‐532‐5p implying the regulation of RUNX3 by miR‐532‐5p.

### miR‐532‐5p targets RUNX3 directly in GC cells

To confirm that RUNX3 is a direct target of miR‐532‐5p, luciferase reporter assay was performed. Wild‐type and mutant RUNX3 3′‐UTR having and lacking miR‐532‐5p binding sequence were cloned into the downstream of firefly luciferase coding region in luciferase reporter vector (Fig. 5F). The relative luciferase activity driven by wild‐type RUNX3 3′‐UTR was reduced about 50% after miR‐532‐5p mimics treatment, but transfection of mutant RUNX3 3′‐UTR restored the relative luciferase by about 90% (Fig. 5G), suggesting that miR‐532‐5p binding sequence was essential for negative regulation of luciferase expression driven by RUNX3 3′‐UTR.

Collectively, our results (Figs 4 and 5) strongly support a direct suppression of RUNX3 by miR‐532‐5p by means of mRNA degradation as well as translational repression.

## Discussion

Recent advances potentiate miRNAs as targets for the diagnosis and antitumour therapies of human cancers, including GC. More and more number of miRNAs were shown to participate in the initiation and progression of GC. Among reported miRNAs, most of them functions as tumour suppressive gene 11, 12, 13, few are oncogenic miRNAs 14. Accordingly, miRNA genes have been characterized as novel proto‐oncogenes or tumour suppressor genes in carcinogenesis 15.

miR‐532‐5p was first reported in cutaneous melanoma 16. The study found miR‐532‐5p was overexpressed in cutaneous melanoma and anti‐miR‐532‐5p inhibited RUNX3 mRNA and protein expression. Another study found that miR‐532‐5p expression was lower in borderline than benign neoplasm and significantly down‐regulated in Her2/neu‐positive ovarian carcinoma 17. However, no information was reported about the function and regulatory mechanism of miR‐532‐5p in other human solid tumour, including GC. The functions of miRNAs are determined by their targets. So, we tried to seek for potential targets of miR‐532‐5p. As listed before, four databases predict that RUNX3 is a candidate.

Previous studies have reported that RUNX3 was absent or lowly expressed in 50–70% primary GC and various GC cells because of hypermethylation of RUNX3 gene promoter, loss of allele heterozygosity and cytoplasmic sequestration 18, 19, 20, 21, but it is unable to interpret the inactivated RUNX3 in those patients without promoter hypermethylation, hemizygous deletion and protein mislocalization. Thus, miRNAs become new candidates to study the mechanism of RUNX3 inactivation. Although inhibition of miR‐532‐5p increased RUNX3 expression in cutaneous melanoma 16, there was still no direct evidence that RUNX3 was a direct target of miR‐532‐5p, as well as the regulation of RUNX3 by miR‐532‐5p in GC. More important, our group have been focusing on RUNX3 in human GC 8, 9, 10, thus we decided to determine the role of miR‐532‐5p and RUNX3 regulation by miR‐532‐5p in GC.

In this study, both transient and stable miR‐532‐5p overexpression promoted cell growth *in vitro* (Figs 1B, C and 2B, C). To investigate the mechanisms underlying cell growth, cell cycle distribution and cell apoptosis were examined. As Figures 1D–H and 2D–H shown, miR‐532‐5p treatment decreased the ratio of cells in G1 phase and apoptotic cells. Previous studies reported that Runx3 could induce p21 and Bim up‐regulation resulting in cell growth inhibition and apoptosis 22, 23, thus we explored the effect of miR‐532‐5p on p21 and Bim expression. As expected, overexpressed miR‐532‐5p decreased the level of p21 and Bim protein (Fig. 4C and F). Overexpression of RUNX3 increased the proportion of cells in G0/G1 phase slightly (Fig. S1) and inhibited proliferation index of GC cells, and our previous studies have showed that overexpressed RUNX3 increased the ratio of apoptosis 10. Thus, we concluded that miR‐532‐5p could inhibit the expression of RUNX3 and its targets p21 and Bim protein, resulting in the relieve of cell growth inhibition and suppression of apoptosis. Accordingly, these results could interpret the enhanced colony foci formation after transient and stable miR‐532‐5p treatment (Figs 1A–C and 2A–C).

To investigate the function of miR‐532‐5p *in vivo*, we injected stable cells into the flank region of nude mice. But, we could not gain the expected subcutaneous xenografts (data not shown). However, we found apparent lung xenografts after injecting stable cells into the tail vein of nude mice compared to control group (Fig. 3A–D). Both increased weight of lungs and more number of tumour foci in lungs confirmed that miR‐532‐5p triggered the invasion of cells from the vein to lungs, cells colonization and growth in lungs. These results can be interpreted by our previous study that overexpressed RUNX3 decreased the number of metastatic nodules in mice lungs 9. High level of miR‐532‐5p and low level of RUNX3 in stable miR‐532‐5p expression group confirmed the stable miR‐532‐5p expression and regulation of RUNX3 by miR‐532‐5p *in vivo*. Although previous study have reported that overexpressed RUNX3 inhibited cell invasion *in vitro* 9, more detailed mechanism underlying the reinforced invasion of GC cells after miR‐532‐5p treatment need to be explored.

Some fresh tissues from primary GC patients were used to determine the expression of miR‐532‐5p and RUNX3, but we could not gain significant results (data not shown). Perhaps the number of samples and the deviation of sampling affected our results. We tried to collect more tissues to determine the expression and role of miR‐532‐5p in GC patients.

Collectively, our present data demonstrated that miR‐532‐5p functions as an oncogenic miRNA in GC cells by targeting RUNX3 at both transcriptional and translational level *in vitro* and *in vivo*. All the results implied that miR‐532‐5p may play an important role in GC development and progression.

## Conflicts of interest

All the authors declared no competing interests.

## Supporting information


**Figure S1** The effect of RUNX3 overexpression on cell cycle distribution. (A) Western blotting analysis of RUNX3 protein expression after transfecting pcDNA 3.1‐RUNX3 plasmid. (B) Cell cycle distribution was examined by flow cytometry after PI staining. (C) Cytometric quantification in (B) showed the ratio of cells in G0/G1, S and G2/M phase. (D) The proliferation index (PI) was calculated using the following equation: PI = (S+G2)/G1, where S, G2 and G1 are the ratio of cells in S, G2/M and G0/G1 phase respectively.
