# Supplementary figures and images for: miRNA‐532‐5p functions as an oncogenic microRNA in human gastric cancer by directly targeting RUNX3
Source: J Cell Mol Med. 2015 Oct 30;20(1):95–103. doi: 10.1111/jcmm.12706 (PMC4717862; doi:10.1111/jcmm.12706)

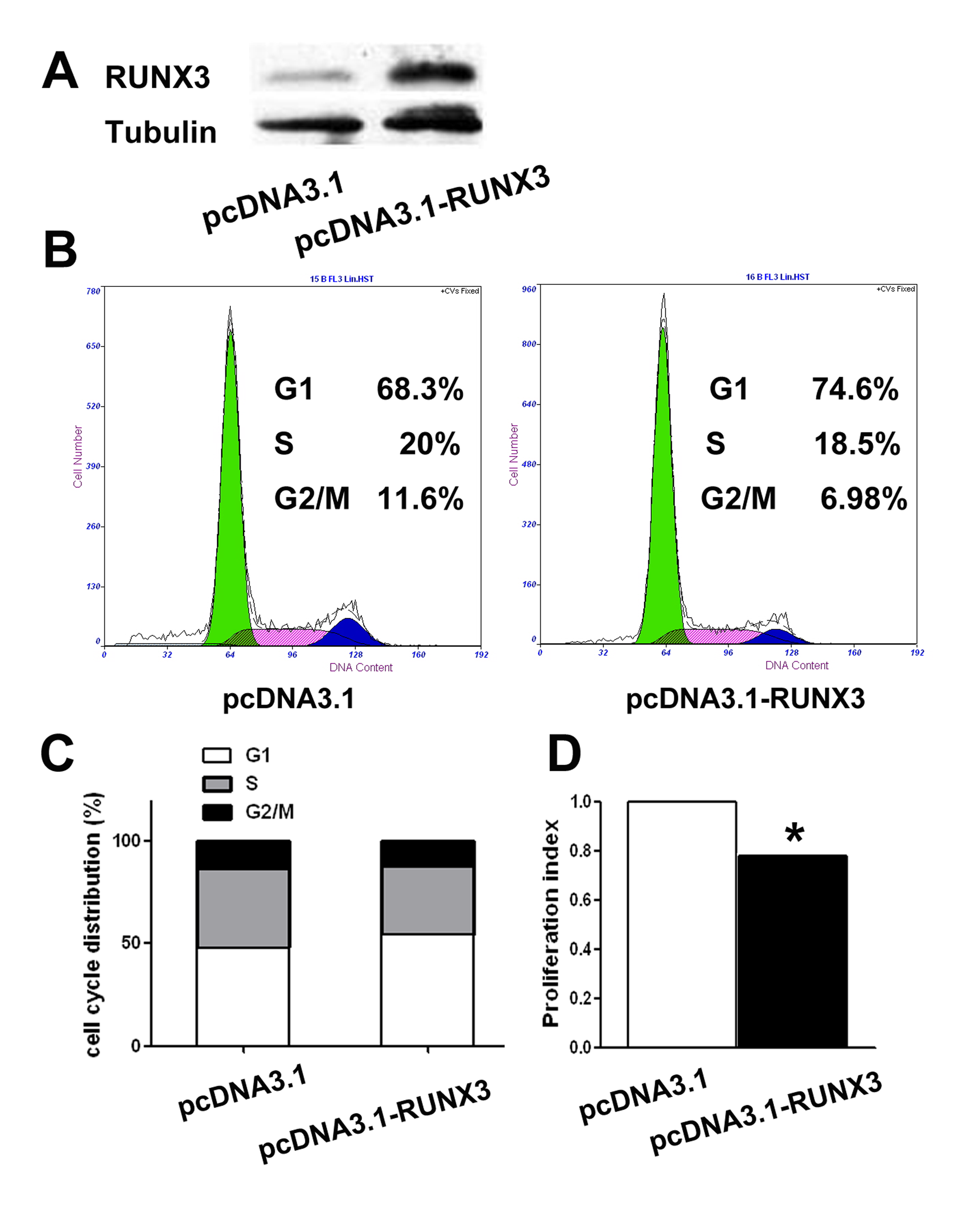

Supplement: Supplementary file 1 — Figure S1 The effect of RUNX3 overexpression on cell cycle distribution. (A) Western blotting analysis of RUNX3 protein expression after transfecting pcDNA 3.1‐RUNX3 plasmid. (B) Cell cycle distribution was examined by flow cytometry after PI staining. (C) Cytometric quantification in (B) showed the ratio of cells in G0/G1, S and G2/M phase. (D) The proliferation index (PI) was calculated using the following equation: PI = (S+G2)/G1, where S, G2 and G1 are the ratio of cells in S, G2/M and G0/G1 phase respectively. [file JCMM-20-095-s001.tif]
